# Mechanism of Action of Secreted Newt Anterior Gradient Protein

**DOI:** 10.1371/journal.pone.0154176

**Published:** 2016-04-21

**Authors:** Kathrin S. Grassme, Acely Garza-Garcia, Jean-Paul Delgado, James W. Godwin, Anoop Kumar, Phillip B. Gates, Paul C. Driscoll, Jeremy P. Brockes

**Affiliations:** 1 Institute of Structural and Molecular Biology, Division of Biosciences, University College London, London, United Kingdom; 2 The Francis Crick Institute, Mill Hill Laboratory, London, United Kingdom; University of Dayton, UNITED STATES

## Abstract

Anterior gradient (AG) proteins have a thioredoxin fold and are targeted to the secretory pathway where they may act in the ER, as well as after secretion into the extracellular space. A newt member of the family (nAG) was previously identified as interacting with the GPI-anchored salamander-specific three-finger protein called Prod1. Expression of nAG has been implicated in the nerve dependence of limb regeneration in salamanders, and nAG acted as a growth factor for cultured newt limb blastemal (progenitor) cells, but the mechanism of action was not understood. Here we show that addition of a peptide antibody to Prod1 specifically inhibit the proliferation of blastema cells, suggesting that Prod1 acts as a cell surface receptor for secreted nAG, leading to S phase entry. Mutation of the single cysteine residue in the canonical active site of nAG to alanine or serine leads to protein degradation, but addition of residues at the C terminus stabilises the secreted protein. The mutation of the cysteine residue led to no detectable activity on S phase entry in cultured newt limb blastemal cells. In addition, our phylogenetic analyses have identified a new Caudata AG protein called AG4. A comparison of the AG proteins in a cell culture assay indicates that nAG secretion is significantly higher than AGR2 or AG4, suggesting that this property may vary in different members of the family.

## Introduction

The first member of the anterior gradient protein family, referred to as XAG2, was identified as a marker of anterior non-neural development in *Xenopus* [1]. The ectopic expression of this protein in embryos induced a supernumerary cement gland, a mucous-secreting element that also expresses XAG2 [2]. It has recently been implicated as a critical secreted signal in *Xenopus* telencephalon formation [3]. Another amphibian AG protein was encountered in the context of salamander limb regeneration [4]. The newt protein nAG was identified as a binding partner of the salamander-specific protein Prod1, a member of the three-finger protein (TFP) superfamily that is GPI-anchored at the cell surface [5]. nAG expression was elevated in Schwann cells of the nerve sheath as peripheral axons regenerate into the newt limb blastema, the regenerative zone at the end of the stump [4]. nAG was subsequently expressed in gland cells underlying the specialised wound epidermis of the blastema. Limb regeneration is normally dependent on the presence of regenerating axons, and it is striking that ectopic expression of nAG could rescue regeneration of a denervated blastema. Recombinant nAG acted to promote S phase entry of cultured newt blastemal cells, possibly by binding to Prod1 on the cell surface, although this requirement has not yet been demonstrated [4].

A mammalian homolog of XAG2, generally referred to as AGR2, is presently the focus of considerable interest in different contexts. It is expressed by secretory epithelia and is upregulated in adenocarcinomas, originally shown in breast cancer cells in culture [6], but subsequently a wide variety of tumour cells including those found in the gastrointestinal tract [7, 8]. The normal development of both intestine and stomach is perturbed by knockout of the mouse AGR2 gene [9–12], leading to an imbalance of differentiated cell types in both organs, and defects in mucous production. It has been implicated in the metastatic phenotype of breast cancer cells, and interacts with alpha dystroglycan and C4.4A, two markers of metastasis [13]. As is the case for Prod1, C4.4A, more commonly known as Ly6/PLAUR domain-containing protein 3 (LyDP3), is GPI-anchored and a member of the TFP superfamily. AGR2 is clearly a significant biomarker for human adenocarcinoma and a potential target for drug discovery [8].

The AG family members are predicted to consist of a N-terminal signal peptide followed by a single thioredoxin domain, as has now been verified by determination of the 3D structures of human AGR2 [14] and human AGR3[15]. Most of the AG proteins, with the exception of the majority of AGR3 orthologues, have a single cysteine residue in the canonical thioredoxin active site. It has been suggested that AG proteins may have an intrinsic protein disulphide isomerase activity, and that this could be implicated in functions dependent on the endoplasmic reticulum (ER) localisation [16]. The AG proteins have a non-canonical Lys/Gln/His-Thr/Ser/Ala/Gly-E-L ER-retention sequence. The models for the function of AGR2 propose a role in attenuating ER stress responses [17], or signalling from the ER to regulate gene expression [16], in particular the amphiregulin gene, which may be critical for mediating the action on cultured adenocarcinoma cells [18], and also the EGF receptor gene [19]. On the other hand, the functions of amphibian and fish AGs, in salamander [4], *Xenopus* [3] and zebrafish [20], involve secretion and extracellular action, and nAG is secreted after transfection of cultured cells [4]. Furthermore AGR2 is found in high concentrations in gastrointestinal mucus [21], and is secreted by human and rat mammary epithelial cells as an O-glycosylated molecule [22]. We address several of these issues, including the activity of secreted nAG, the dependence on the single Cys residue in the thioredoxin active site, and the variation of ER retention versus secretion in different AG proteins.

## Materials and Methods

### Site-directed mutagenesis and plasmids

The newt wild type *nag* sequence was amplified using oligos (Forward 5’-GCGGCTAGCATCGCTCAACATGGTGAA-3’, Reverse 5’-CGCGTCAATTCTTCGCTCACA-3’) on newt blastemal cDNA and cloned between the *Nhe1* and *EcoR1* sites of the vector pCI-neo (Promega) to give pnAGWT. The cysteine at position 72 was mutated to a serine using oligos (Forward, 5’-CACAGAGATGACTCTCCACACTCTCAGGCTTTGAAGAAAG-3’, Reverse, 5’-CTTTCTTCAAAGCCTGAGAGTGTGGAGAGTCATCTCTGTG-3’) or an alanine (Forward, 5’- CACAGAGATGACGCTCCACACTCTCAGGCTTTGAAGAAAG-3’ Reverse, 5’-CTTTCTTCAAAGCCTGAGAGTGTGGAGCGTCATCTCTGTG-3’) using the Quikchange site-directed mutagenesis kit (Stratagene) to create mutant clones nAG-C72S and nAG-C72A respectively.

Myc tags were added at the 3'end of these inserts by PCR using oligos 690 (Forward, 5’-CACAGAGATGACGCTCCACACTCTCAGGCTTTGAAGAAAG-3’, Reverse, 5’-CGCTCTAGAAATCAGATCCTCTTCTGAGATGAGTTTTTGTTCCAGTTCAGTTTTCAGAAGTTTGAGTGCT-3’) and the resultant fragments were cloned into the *EcoRI* and *XbaI* sites of pCI-neo expression vector to give 3 myc+25 constructs. We found that the translation product used a vector stop site, resulting in an expressed protein product with an additional 25 amino acid residues (ISRVDPGGRFPLVRVNASSRHDKIH) at the C terminus which are derived from a sequence in the vector. This appendage proved to be beneficial for the recovery of the protein from the conditioned medium. For derivation of constructs without these additional amino acids, the wild type and mutant constructs were amplified with oligos 690 and primer 5’-CGCTCTAGACTACAGATCCTCTTCTGAGATGAGTTTTTGTTCCAGTTCAGTTTTCAGAAGTTTGAGTGCT-3’, and the resultant fragments were cloned into the *EcoRI* and *XbaI* sites of pCI-neo vector to give 3 myc-tagged constructs.

The plasmid pEGFP-N2 (Clontec) was used as a negative control in parallel transfections. In some cases a construct pERFP-N2 was used, where the GFP (green fluorescent protein) sequence in pEGFP-N2 is swapped for RFP (red fluorescent protein).

### Animals, cell culture and transfection

The animal maintenance and procedures of the experiments were approved by the Home Office, UK. Adult newts were obtained from Charles D. Sullivan & Co. TN, USA, and maintained in aquariums at 25°C. The animals were fed with live blood worms throughout the experimental period. Bilateral forelimb amputation was performed under anaesthesia (0.1% tricaine) and the newts were allowed to regenerate. Isolation and culture of limb blastema cells has been described previously [23]. Briefly, the limb blastemas at mid-cone stage of growth (12–14 days after limb amputation) were collected, and the blastema cells were dissociated non-enzymatically from the mesenchyme in amphibian cell culture medium. The cells were plated on to collagen-coated (Type I, Sigma C8919) 96 well microplates and maintained at 25°C with 2% CO_2_ supply. The cell density varied from 1000–2000 cells per well in various batches. The assay for S phase entry have been described [4]. In brief, the cells were cultured for 72 h, and the medium was changed to include the various additives, either rabbit IgG 683 to Prod1 [5], or affinity purified control rabbit IgG (Sigma 15006), and 10 μM BrdU, followed by a 96 h incubation prior to fixation and antibody staining. The microwells were scanned using a Zeiss Axiovert 200 microscope controlled through Axiovision (Carl Zeiss, Version 4.8.2) software. The images were stitched and exported to Image Pro Plus (Media Cybernetics) for processing and cell counting [4]. Cos7 and HEK 293T cell lines were purchased from ATCC and Invitrogen respectively, and cultured under standard mammalian cell culture protocol in Dulbecco’s Modified Eagle Medium containing 10% fetal bovine serum. The cells were seeded on to 30 or 60 mm culture wells coated with poly(ethyleneimine) solution (1 μg/mL; Sigma, P3143), and transfected at 80% density with nAG-expressing pCIneo plasmids using Lipofectamine 2000 as described [24]. The transfection efficiency in Cos7 cells varied from 40–60%, whereas HEK 293 cells attained 80% transient expression.

### Cell lysates, conditioned medium and western blotting

For the experiments on the nAG mutants, cells were lysed in a buffer containing 1% NP40, 0.15 M NaCl, 1 mM Na_3_VO_4_, 0.05 M Tris-HCl pH 7.5 supplemented with protease inhibitors (Complete Cocktail Tablets, Roche). For the experiments on transfection of AG family members, the cells were lysed in a buffer containing 0.15 M NaCl, 1 mM EDTA, 0.1% SDS, 1% NP40, 1% sodium deoxycholate, 10 mM Tris-HCl pH 7.8, supplemented with protease inhibitors. Cell lysates were centrifuged at 5000 *g* for 10 min, and solubilised in sample buffer prior to analysis by electrophoresis.

After incubating Cos7 or HEK 293T cells for 48 or 72 h, the culture media were collected, adjusted to 10 mM HEPES pH 7.4 and centrifuged at 180 *g* for 10 min prior to loading onto Vivaspin ultrafiltration units with a 10 kD exclusion limit. The concentrated media samples were solubilised in sample buffer. Cell lysates and concentrated conditioned media samples were analysed by electrophoresis on precast Novex 12% Bis-Tris SDS polyacrylamide gels, and transferred to nitrocellulose by standard methods prior to antibody reaction. For detection of nAG we used the polyclonal rabbit antibody 224 [5] at a dilution of 1:500 and fluorescent secondary goat anti-rabbit conjugated to IR dye emitting at 800 nm (Rockland). For determination of absolute levels of nAG and mutants, we used a protein standard of nAG with C-terminal hexa-histidine tag, produced in insect cells using the baculovirus expression system. For detection of AG4 and AGR2 we used rabbit anti-human AGR2 (Sigma) made against residues 108–157 in the sequence of AGR2, and detected with the same secondary antibody. The blots were analysed in a LICOR Odyssey scanner, and the nAG band intensities were multiplied by the appropriate dilution factors to give estimates for the total secreted and cell-retained protein in the culture dishes. Blots were routinely co-stained with mouse anti-actin (Sigma) and goat anti-mouse emitting at 600 nm. The actin signals were not used in the determination of percentage secretion (since actin is not secreted), but in comparison of lysates to ensure that the nAG, AG4 and AGR2 proteins were expressed at comparable levels and not subject to marked differences in expression after transfection.

### Phylogenetic analyses

Tblastn was used to mine nucleotide sequences from databases including NCBI, Ensembl, Sal-site [25], and newt transcriptomes [26, 27] (S1 Table). Multiple sequence alignments were calculated using MUSCLE [28] and MAFFT [29] and inspected and modified using Jalview [30] and ClustalX [31]. GTR+G+I was selected as an appropriate substitution model using ModelGenerator [32]. Trees were computed by maximum likelihood using PhyML3 [33] with 1000 bootstrap replicates and by Bayesian inference with MrBayes 3.2 [34]. For Bayesian calculations convergence was assessed using AWTY [35]. Trees were visualised and figures were prepared using Dendroscope [36].

## Results

### Activity of the nAG protein on newt blastemal cells

**Fig 1:**
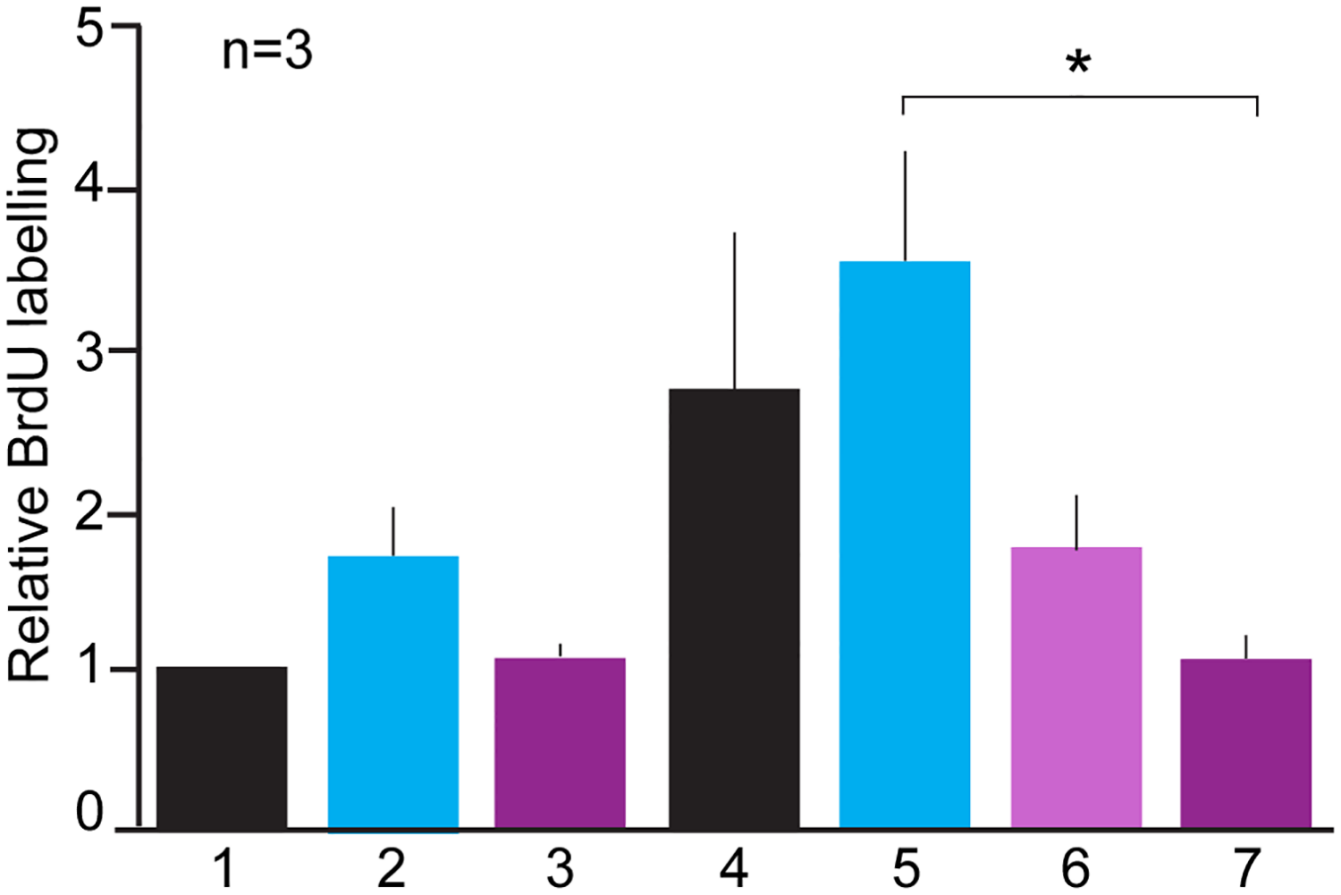
Antibody inhibition of nAG-induced S phase entry. Newt limb blastemal cells were cultured in microwells as described, and exposed to either conditioned medium from Cos7 cells transfected with control RFP plasmid (1–3), or nAG plasmid (4–7). Control rabbit IgG or affinity-purified rabbit IgG to Prod1 was added to the wells as follows:(1) control medium (2) control rabbit antibody, 20 μg/mL (3) anti Prod1, 20 μg/mL (4) nAG-containing medium, (5) control rabbit antibody, 20 μg/mL (6) anti Prod1, 10 μg/mL (7) anti Prod1, 20 μg/mL. Note the inhibition of nAG activity by anti Prod1 (* P<0.5) but not by control. Data from three independent transfections each normalised to the value for the RFP control, expressed as mean ± SD. The data were analysed by One-way Analysis of Variance (ANOVA) followed by Tukey’s multiple comparison test.

In order to investigate the activity of recombinant nAG, mammalian Cos7 cells were transfected with plasmids expressing either nAG or red fluorescent protein (RFP) as control. The conditioned medium was concentrated and added to microwell cultures of newt limb blastemal cells for 3 days. Affinity purified rabbit IgG against newt Prod1, or control IgG, was added to some wells, and the effect on S phase entry was determined by incorporation of bromodeoxyuridine. The results of assays from three independent preparations of blastemal cells were normalised and are shown in Fig 1. The activity of nAG medium was inhibited to the level of control medium by inclusion of antibody to Prod1 at 20 μg/mL (Fig 1, Lanes 4, 6 and 7), while control IgG had no inhibitory action (Fig 1, Lane 5). The background activity of RFP control medium was not inhibited by addition of either antibody (Fig 1, Lanes 1, 2 and 3). These results are consistent with the hypothesis that the stimulatory activity of nAG on the newt blastemal cells is mediated by interaction with its binding partner Prod1.

### Expression and activity of Cys72 mutations

**Fig 2:**
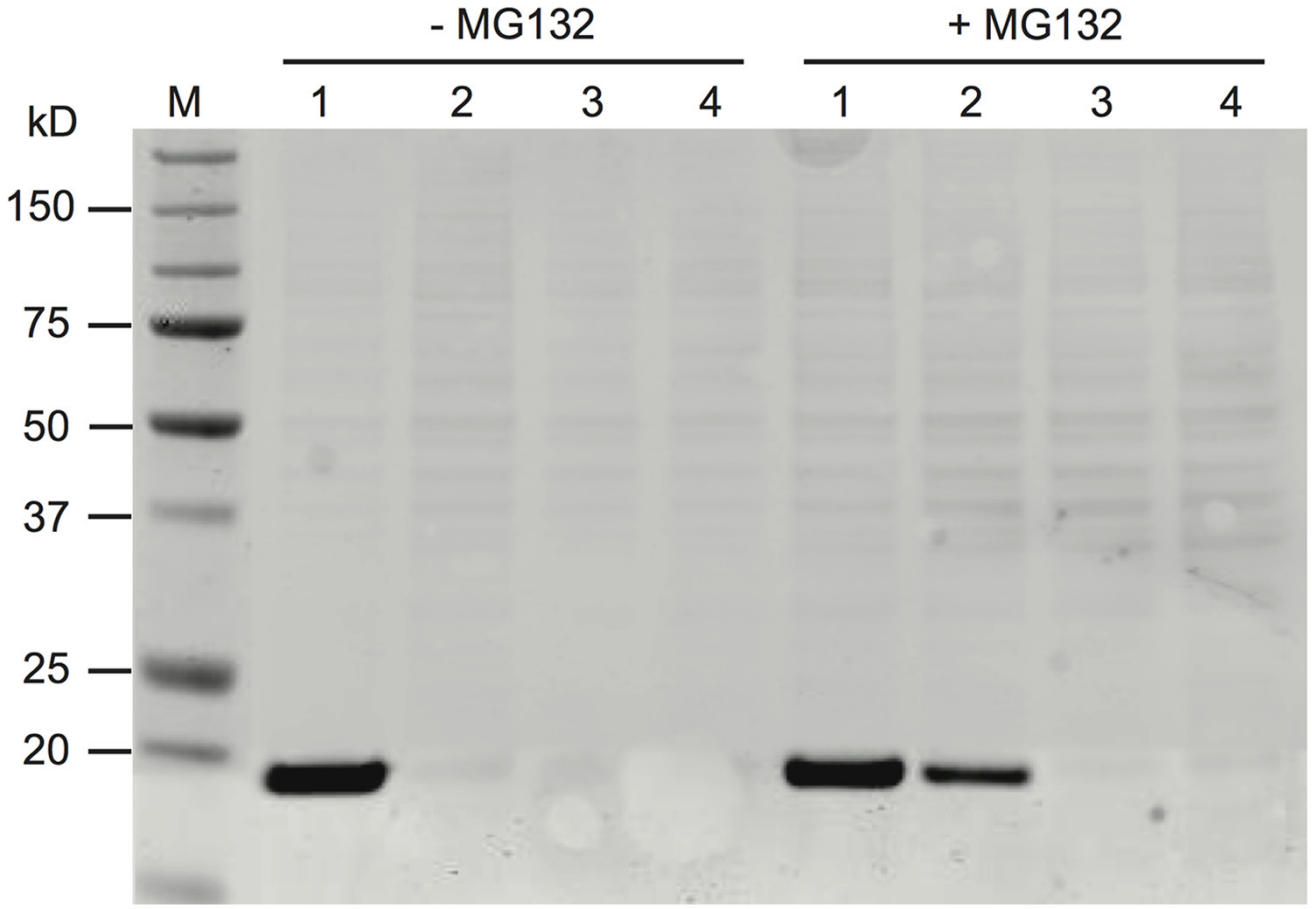
Degradation of Cys72 mutants of nAG. Cos7 cells were transfected with plasmids expressing (1) wild-type nAG, (2) C72A nAG, (3) C72S nAG, (4) GFP control, and cultured in the presence or absence of 50 μM MG132 as shown. The conditioned medium was collected, concentrated and analysed by western blotting as described in the Materials and Methods.

Plasmids that encode wild-type nAG, or mutants of the protein at Cys72, were transfected in parallel into Cos7 cells or 293T cells in culture. After 2 days the conditioned medium and cell lysates were prepared for analysis by gel electrophoresis and western blotting. The wild-type protein was readily detectable in the conditioned medium of both cell types as an immunoreactive band at 17 kD (Fig 2). The mutant proteins were barely detected in both the conditioned medium (Fig 2) and the lysates. Quantitative analysis of the C72A band (with reference to a wild-type nAG band) indicated that the level of protein in the medium was less than 10% of wild type. The low level of expression of the mutant proteins was observed after transfection of both mammalian cell types. This effect was partially relieved for C72A by culturing the cells in the presence of the proteasome inhibitor MG132 (Fig 2). It was not possible to assay such conditioned medium for activity on cultured blastemal cells because the cells were very sensitive to the inhibitor. Thus the mutation of the Cys72 residue destabilises the nAG molecules and leads to their degradation, probably via the proteasome.

**Fig 3:**
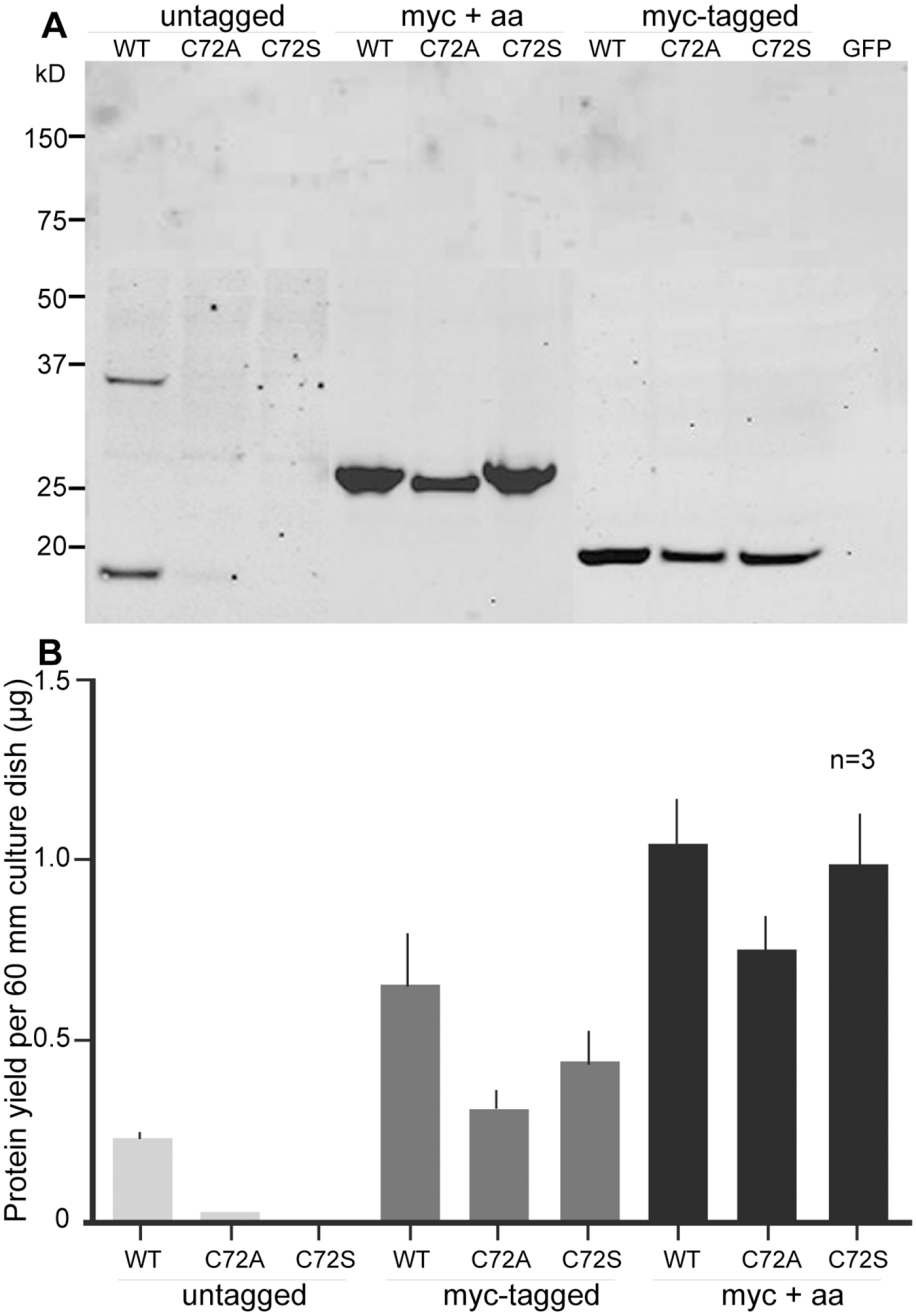
Expression of nAG proteins with C terminal extensions. (A) Wild-type and mutant nAG proteins, with or without (untagged) additional residues at the C terminus, were expressed in Cos7 cells and the conditioned medium was analysed as described in Fig 2. Note that the myc-tagged samples were run on a separate gel to the other samples, and the four lanes were joined to the right hand side. GFP refers to control GFP-transfected cells. (B) The yield of nAG proteins in the medium was quantitated by analysis of western blots for three independent transfections. Note that the largest yield for each of the three proteins is obtained with the myc+25 extension.

**Fig 4:**
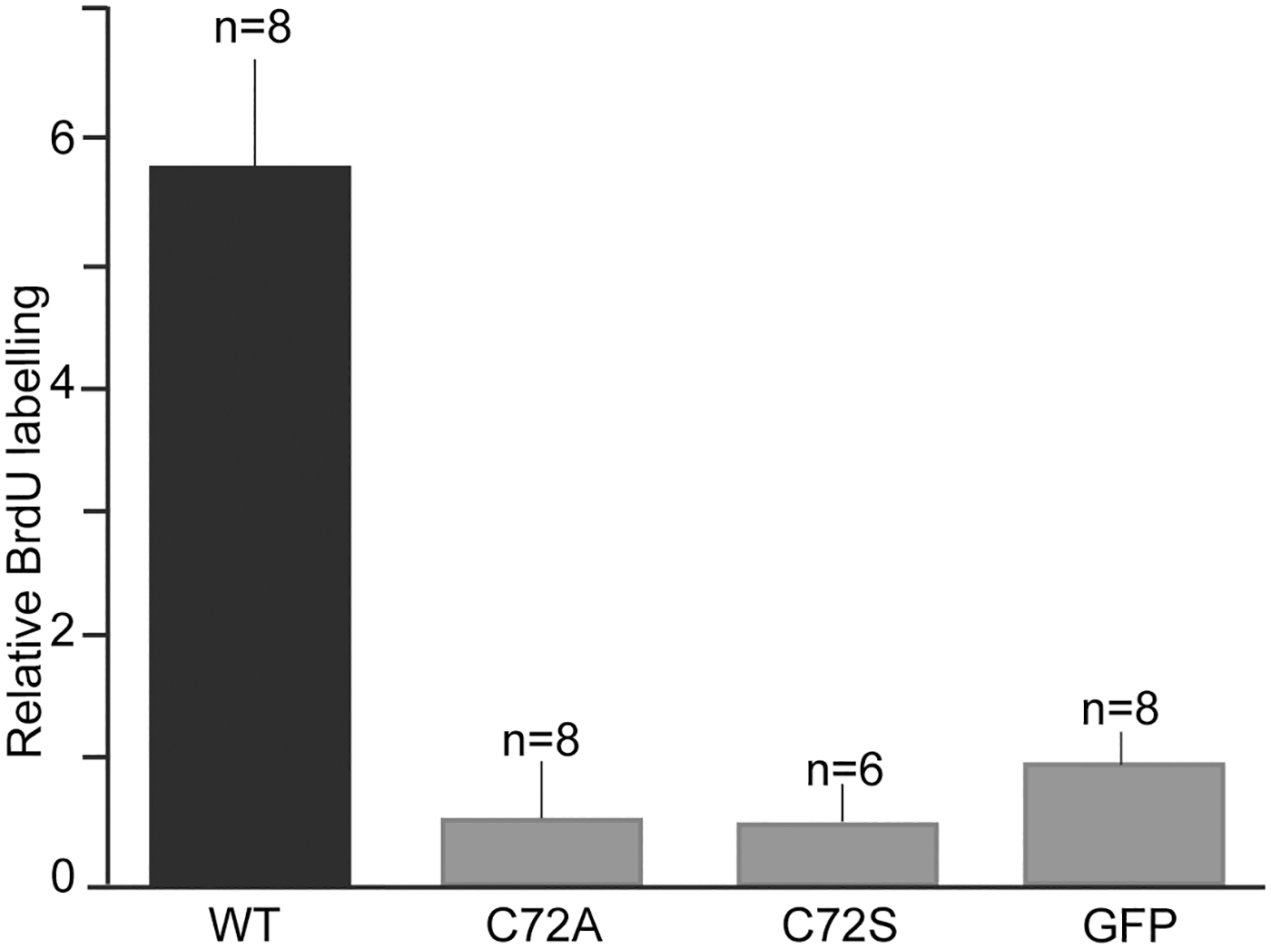
Mutation of Cys72 leads to loss of activity. Cos7 cells were transfected with constructs expressing wild-type (WT), C72A, or C72Seither without a tag (untagged), with a myc-tag plus (myc + 25) or myc-tag minus (myc-tag without 25 aa). The conditioned media were analysed by western blotting and equivalent amounts of protein were added to blastemal cells growing in microwells. The data are given for the number (n) of microwells from 3 (in case of C72S) or 4 (other proteins) independent preparations of blastemal cells, normalised to the value for GFP.

When a myc epitope was introduced at the C terminus of the three nAG protein constructs, they were recovered at significantly higher levels in the conditioned medium of transfected Cos7 cells, even in the case of the wild-type protein (Fig 3A and 3B). We made a chance observation that, when the proteins were further extended for 25 residues after the myc tag, the levels in the medium were even higher, and there was little difference in yield between the wild-type and mutant protein variants (Fig 3A and 3B). The wild-type nAG and its extended variant (myc+25) had comparable activity in promoting S phase entry of cultured blastemal cells, and thus we were able to compare the activities of the extended versions of the three proteins at the same concentration. The data for three or four assays on independent preparations of cells have been combined and are shown in Fig 4. The magnitude of stimulation by the extended wild-type protein was comparable to that reported for the normal protein [4]. The mutation of Cys72 to either Ala or Ser leads to no detectable activity over the background of control (GFP plasmid-transfected) medium. These data suggest that the Cys72 residue is required for the proliferation-inducing activity of nAG.

### Retention and secretion of AG proteins

**Fig 5:**
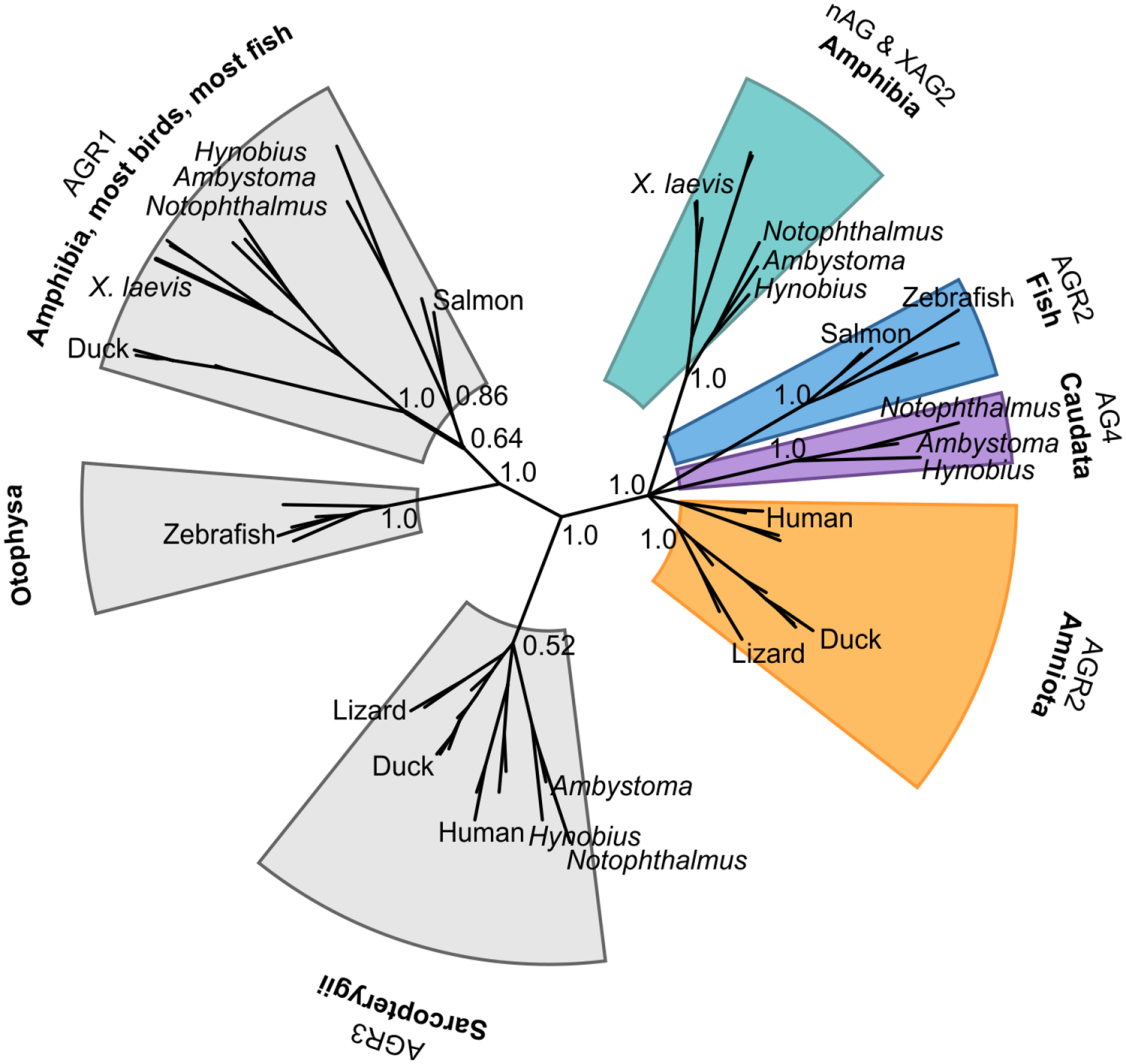
Representative phylogenetic tree highlighting the species distribution of selected anterior gradient genes. For clarity only relevant support values are shown. Note that proteins belonging to the AG4 cluster have, so far, only been found in salamanders while the nAG and XAG2 grouping contains both salamander and anuran sequences. The tree was calculated with MrBayes 3.1 using likelihood model parameters: nst = 6; rates = invgamma. The calculation was run for 10 million generations, sampling every 500 generations; the initial 5,000 trees were discarded. An equivalent clustering of sequences was obtained using a maximum-likelihood approach as implemented in Phyml3 using the LG+G+I model and 1000 bootstrap replicates.

Through mining of publicly available transcriptomic and genomic databases we have identified a novel group of Caudata (salamander) AG proteins, which we have called AG4. In molecular phylogenetic analyses (this work and [20]), AG proteins are vertebrate-specific and cluster in four groups each of which has a different distribution of vertebrate classes (Fig 5 and S1 Table) and a characteristic thioredoxin active site/C-terminus signature (S1 Fig).

The AGR1 group can be identified by its active site sequence Cys-Pro-His/Tyr-Ser and typically a His/Lys-Thr/Ser-Glu-Leu C-terminal region. This group includes fish, turtle, bird and amphibian sequences. Not all bird genomes contain an AGR1 gene, but its presence in the basal clade Palaeognathae (flightless birds) suggests that the gene was subsequently lost in certain lineages (e.g. Phasianidae). The AGR1 proteins found in Otophysa fish such as zebrafish and the cave fish *Astyanax mexicanus* are markedly different from the rest and can be recognised by a six-residue C-terminal extension not observed in any other AG protein. The AGR3 gene is only present in lobe-fin fishes and tetrapoda (Sarcopterygii). The signature sequences are Cys-Gln-Tyr-Cys/Ser for the active site and Gln-Thr/Ser/Lys-Glu-Leu at the C terminus.

The cluster that includes human AGR2 contains one gene per species from fish to mammals, except for Caudata species which have two genes in this group, nAG and AG4. The signature sequences for the group are Cys-Pro-His-Ser in the active site, and a Lys-Thr/Ser-Glu-Leu C terminus. At an amino acid level, AG4 sequences are slightly more similar to mammalian AGR2 than nAG (e.g. 75% vs 69% identity between human AGR2 and *Notophthalmus* AG4 and nAG, respectively). Intriguingly, the AG4 sequences are also the only ones that diverge from the rest of cluster with respect to their thioredoxin active site/C-terminus signature (S1 Fig).

Despite the fact that they possess an identical ER-retention signal (Lys-Thr-Glu-Leu), human AGR2 is apparently largely retained in the ER as detailed in the Introduction, while nAG is apparently secreted. We hypothesised that, despite its divergent C-terminal sequence, AG4 might behave in a similar manner to AGR2 and also be an ER-retained protein. In order to provide an initial test of this hypothesis, we transfected 293T cells with constructs expressing wild-type nAG, or human AGR2, or newt AG4. In each case the conditioned media were collected after 3 days, concentrated, and analysed by western blotting in parallel with the cell lysates. Commercial antibodies to a 50-residue section of AGR2 reacted strongly with AG4 but not with nAG, consistent with AG4 and AGR2 being more closely related to each other than to nAG. The signals from the western blots were expressed as the percentage of secreted AG protein in each dish, as detailed in the legend to Table 1 and the methods section. These values were averaged over four independent transfections (Table 1). The proportion that was secreted for AG4 was approximately 20-fold lower than that for nAG, while that for AGR2 was approximately 6-fold lower. Therefore, there was a significant difference in secretion between newt AG4 and nAG. It was not possible to repeat this analysis in cultured salamander cells, as the level of expression after transfection proved too low for analysis by western blotting.

**Table 1:** Differential secretion of AG family members by cultured cells.

Protein	% secreted protein
nAG (newt)	10.6 ± 2.7
AGR2 (human)	1.65 ± 0.5
AG4 (newt)	0.5 ± 0.1

HEK293T cells were transfected in parallel with plasmids expressing the wild-type (untagged) proteins nAG, human AGR2 or newt AG4. After 72 h the conditioned medium and cell lysate for each dish was processed and analysed by western blotting as described in the Materials and Methods section. The results are expressed as the percentage of the total immunoreactive AG protein in the dish that is secreted, with the results (mean ± SD) averaged over four independent transfections.

## Discussion

Mutation of the single cysteine residue in nAG led to protein degradation after transfection, possibly by the ERAD pathway. This loss was inhibited by extensions of the sequence at the C terminus, probably by interfering with the retrieval to the ER by the KDEL receptors, thus leading to more rapid transit through the secretory pathway. This serendipitous finding allowed us to obtain enough of the mutant nAG proteins to establish that the cysteine residue is essential for the S phase entry activity of the extended wild-type protein. The secreted protein in the conditioned medium predominantly migrated on non-reducing SDS gels as a monomer band, so it is likely that the sulfhydryl group was not involved in disulfide-bonded homodimer formation. This finding was in agreement to what had been reported for recombinant human AGR2 [14], although results using cell lysates suggested otherwise [17]. It is possible that the mechanism of action could involve formation of a heterodimeric disulfide-bonded intermediate with Prod1 on the cell surface, comparable to that observed between AGR2 and mucin [10], although the presence of such a covalent bond has been questioned [21]. Although, as in the case of others [14], we have been unable to detect any PDI activity using conventional biochemical assays, the fact that the proliferation-inducing activity of nAG is abrogated in the absence of Cys72 strongly suggests that this residue is able to form a mixed disulfide bond, perhaps in the presence of other proteins or small molecules yet to be found.

The hypothesis that secreted nAG acts on blastemal cells via the GPI-linked Prod1 receptor [4] is supported by our data showing that the S phase entry activity of nAG is inhibited by an antibody to Prod1. It has recently been reported that mouse AGR2 acts via the orphan membrane receptor C4.4A in models of pancreatic adenocarcinoma [37]. The administration of antibodies against C4.4A reduced growth and metastasis in mice [37]. It is possible that the ability of AG proteins to signal through GPI-linked TFP superfamily members is conserved through evolution, but the mechanism of action is a subject for future study.

Previous studies have highlighted that some members of the AG family are not present in amniotes, and that these proteins may contribute to the enhanced regeneration of appendages observed in amphibians [38]. Our results are broadly consistent with this suggestion but, as our phylogenetic analysis encompassed a wider variety of sequences and species, we found two significant differences: first, it is the cluster encompassing nAG and XAG2 that only contains amphibian sequences, and second, the AGR1 cluster does include amniote, but not mammalian, sequences and it may consist of more than one paralogue. The Caudata AG4 members are closest in amino acid sequence identity to Amniota AGR2 but their dissimilar active site and ER retention signals suggest that they are experiencing distinct selective pressures to AGR2, perhaps due to the co-existence of multiple AG proteins that might have partially overlapping functions. We speculate that the importance of nAG for regeneration in Caudata, and XAG2 for development in Anura, reflects the evolution of a branch where the AG protein is secreted and acts in the extracellular space (green in Fig 5). The AG4 branch (purple in Fig 5) could maintain the essential ER-related functions proposed for AGR2. It is interesting that the ER-retention signal for AG proteins varies across the different families, suggesting that novel activities might evolve readily by modulating the cellular localisation and/or the degree of retention of these proteins in the ER. Much remains to be understood about the diversification of the AG protein family, and its roles in development, regeneration and cancer.

## Supporting Information

S1 Fig Multiple sequence alignment of representative anterior gradient proteins. The positions are coloured by chemical properties and conservation as defined by the ClustalX scheme.
(TIF)

S1 Table List of anterior gradient nucleotide sequences. The details of anterior gradient nucleotide sequences used in the phylogenetic analyses with their accession codes are listed here.
(XLS)
