# Supplementary figures and images for: Mechanism of Action of Secreted Newt Anterior Gradient Protein
Source: PLoS One. 2016 Apr 21;11(4):e0154176. doi: 10.1371/journal.pone.0154176 (PMC4839744; doi:10.1371/journal.pone.0154176)

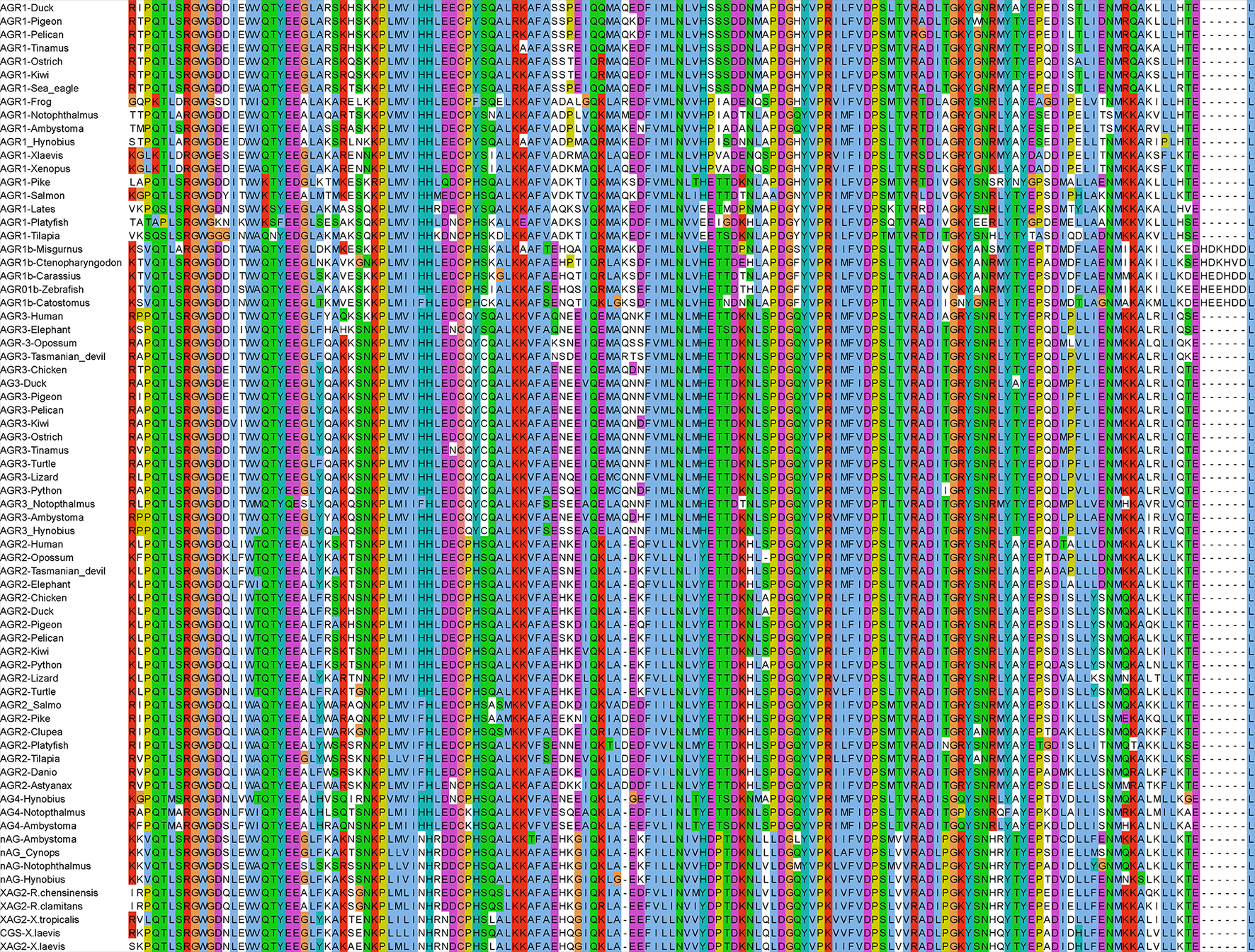

Supplement: S1 Fig — The positions are coloured by chemical properties and conservation as defined by the ClustalX scheme. (TIF) [file pone.0154176.s001.tif]
